# Second molar impaction associated with lip bumper therapy

**DOI:** 10.1590/2176-9451.19.6.099-104.oar

**Published:** 2014

**Authors:** Helder Baldi Jacob, Shawn LeMert, Richard G. Alexander, Peter H. Buschang

**Affiliations:** 1 Texas A&M University Baylor College of Dentistry, Postdoc resident in Orthodontics, Texas A&M University Baylor College of Dentistry; 2 Texas A&M University Baylor College of Dentistry, PhD in Dentistry, Texas A&M University Baylor College of Dentistry; 3 Texas A&M University Baylor College of Dentistry, Department of Orthodontics, Professor, Department of Orthodontics, Texas A&M University Baylor College of Dentistry; 4 Texas A&M University Baylor College of Dentistry, Professor, Texas A&M University Baylor College of Dentistry

**Keywords:** Impacted tooth, Unerupted tooth, Molar tooth, Interceptive Orthodontics

## Abstract

**INTRODUCTION:**

Although lip bumpers (LBs) provide significant clinical gain of mandibular arch perimeter in mixed-dentition patients, orthodontists are reluctant to use them due to the possibility of permanent second molar eruptive disturbances.

**OBJECTIVE:**

The present study was conducted to assess second molar impaction associated with the use of LBs, and to investigate how they can be solved.

**MATERIAL AND METHODS:**

Lateral and panoramic radiographs of 67 patients (34 females and 33 males) were assessed prior (T_1_) and post-LB treatment (T_2_). LB therapy lasted for approximately 1.8 ± 0.9 years. Concomitant rapid palatal expansion (RPE) was performed in the maxilla at LB treatment onset. Impaction of mandibular second molars was assessed by means of panoramic radiographs in relation to the position of first mandibular molars. Horizontal and vertical movements of first and second molars were assessed cephalometrically on lateral cephalometric radiographs based on mandibular superimpositions.

**RESULTS:**

Eight (11.9%) patients had impacted second molars at the end of LB therapy. Two patients required surgical correction, whereas five required spacers and one patient was self-corrected. Mandibular first molar tip and apex migrated forward 1.3 mm and 2.3 mm, respectively. Second molar tip showed no statistically significant horizontal movement.

**CONCLUSION:**

Although LB therapy increased the risk of second molar impaction, impactions were, in most instances, easily solved.

## Abstract (pt)

**INTRODUÇÃO:**

embora placas labioativas (PLAs) promovam um ganho clínico significativo no perímetro da arcada inferior em pacientes com dentição mista, ortodontistas são relutantes em usá-las devido a possibilidade de problemas eruptivos dos segundos molares.

**OBJETIVO:**

o presente estudo avaliou a impacção dos segundos molares associada ao uso das PLAs e como a impacção pôde ser resolvida.

**MÉTODOS:**

radiografias cefalométricas lateral e panorâmica de 67 pacientes (34 do sexo feminino e 33 do masculino) foram avaliadas antes (T_1_) e após (T_2_) o tratamento com PLAs, que durou aproximadamente 1,8 ± 0,9 anos. Expansão rápida do palato (ERP) foi usada na maxila no início do uso da PLA. Usando as radiografias panorâmicas, a impacção dos segundos molares inferiores foi avaliada relativamente à posição dos primeiros molares mandibulares. Os movimentos horizontais e verticais dos primeiros e segundos molares inferiores foram avaliados com base em sobreposições de estruturas estáveis da arcada inferior por meio das radiografias laterais.

**RESULTADOS:**

oito (11,9%) pacientes apresentaram impacção dos segundos molares mandibulares ao final do tratamento com PLA; dois pacientes tiveram de recorrer à intervenção cirúrgica para a correção da impacção, cinco tiveram a correção da impacção usando apenas espaçadores e um apresentou autocorreção da impacção. A coroa e o ápice do primeiro molar inferior migraram 1.3mm e 2,.3mm, respectivamente, para mesial. O segundo molar não mostrou movimento horizontal significativo.

**CONCLUSÃO:**

embora o tratamento com PLA aumente o risco de impacção do segundo molar mandibular, as impacções, na maioria das vezes, podem ser facilmente corrigidas.

## INTRODUCTION

Tooth size-arch length discrepancy (TSALD) is a common malocclusion. Approximately 31% of North American adolescents have more than 4 mm mandibular irregularity,^1^ while approximately 40% of adults have irregularities greater than 3.5 mm.^2^ Depending on facial balance, crowding can be treated either by reducing tooth mass or by increasing arch size. For mild-to-borderline moderate TSALD, lip bumpers (LBs) are commonly used as an adjunctive treatment to gain space in mixed-dentition patients.

By maintaining leeway space and increasing arch width, LBs have proved an effective and relatively stable treatment approach.^3^^,^ 4 ^,^ 5 LBs are inserted into buccal tubes cemented to first permanent molars, maintained in front of and away from lower anterior teeth and activated by lower lip pressure. Because they keep lower lip and buccal musculature away from mandibular teeth, LBs disrupt equilibrium which causes the crowns to move in buccal direction.^3^^,^ 6 ^,^ 7 ^,^ 8 The therapeutic effects of LBs include increase in arch width, particularly in premolar and molar regions, and an increase in arch depth associated with proclination of incisors and distal tipping of molars.^3^^,^ 6 ^-^ 9

One of the main reasons orthodontists are reluctant to use LBs is their potential to produce permanent second molar disturbances of eruption. LBs - especially those with relatively thick shields of acrylic from canine to canine - tend to distally tip mandibular first molars.^6^^,^ 8 Since LBs are most effective when treatment is initiated in the mixed dentition, distal molar tipping could prevent normal eruption of second molars. Ferro et al^10^ recently showed that mixed dentition patients treated with LBs are more likely to exhibit second molar impaction (7%) and ectopic eruption (16%) than untreated patients for whom eruptive disturbances have been reported to range from 0.1 to 2.5%.^10^^-^ 15 Despite being crucial to understand the effects over second molars, movement of mandibular first molars during LBs therapy has not been properly studied. 

The present study was conducted to assess second molar impaction with the use of LBs. The aims were to: 1) to assess the likelihood of second molar impaction; 2) to establish tooth movement during LB wear; 3) to describe how second molar impaction was solved.

## MATERIAL AND METHODS

This observational retrospective, longitudinal, study used lateral and panoramic radiographs of 67 patients (34 females and 33 males) with a pretreatment (T_1_) mean age of 10.6 ± 1.3 years and a post-LB (T_2_) mean age of 12.3 ± 1.2 years. All patients were treated by the same orthodontist.

Patients were selected based on the following criteria:

" Lateral and panoramic radiographs had to be available at the start (T_1_) and end (T_2_) of LB therapy.
" Patients should have unerupted second permanent molars.
" Patients should be treated under the same rapid palatal expansion (RPE)/LB therapy protocol.

In the maxillary arch, patients were treated with Hyrax RPE. The jackscrew was placed at first molars as high into the palate as possible. Patients were advised to turn the screw once a day (0.25 mm) for four weeks. After adequate expansion had been achieved, the RPE screw was locked in position with composite resin and left in place for approximately six months.

**Figure 1.**
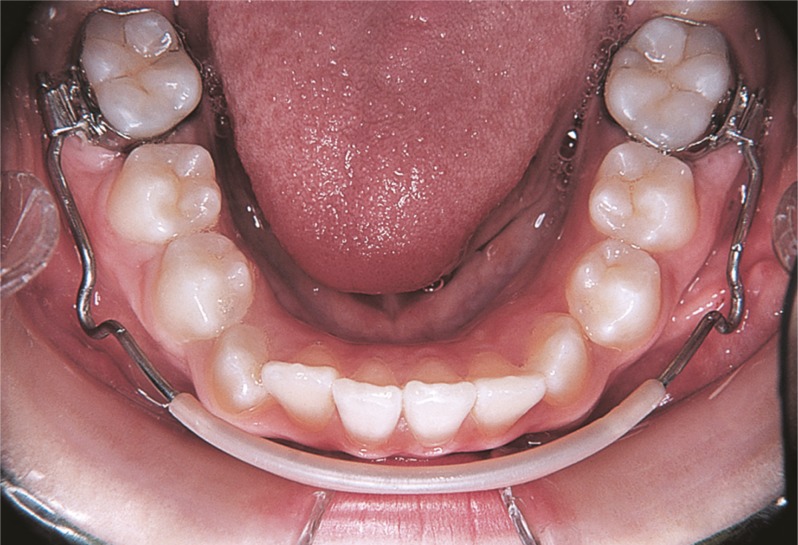
Occlusal view of pre-fabricated lip bumpers with an acrylic shield extending from canine to canine and fitted on molar tubes.

While maxillary expansion was started, LBs were used in the mandibular arch. LBs were pre-fabricated with an acrylic shield extending from canine to canine (Fig 1). Each LB was adjusted so that the acrylic shield was 2-3 mm away from the labial surface of lower incisors and 4-5 mm away from the facial surfaces of buccal segments. The LB was activated to provide approximately 3-4 mm of expansion at the molar region. It was adjusted at three to four week intervals. The active phase of LBs was approximately 10 months after which the appliance was maintained until full fixed appliance was placed.

Impaction of second molars was diagnosed based whether or not eruption into full occlusal contact with their antagonist had been interrupted. Eruption can fail totally or partially due to physical barrier in the path of eruption.^16^ According to Raghoerbar et al,^16^ impacted molar shows greater angulation between the long axis and normal eruption path. Diagnosis was based on the position of mandibular second molars at the end of lip bumper therapy, with consideration given to the position of first mandibular molars as well as the eruptive status of maxillary molars. Also, root length of mandibular second molars was assessed to differentiate between the possibility of delayed eruption and impaction.

To assess the movements of first mandibular molars and central mandibular incisors, mandibular superimpositions were performed using natural reference structures.^17^ Radiographic tracings were oriented on the basis of: 1) anterior contour of the chin; 2) inner contour of the cortical plate at the lower border of mandibular symphysis; 3) distinct trabecular structures in the mandibular symphysis; 4) contour of the mandibular canal; and 5) lower contour of third molar tooth germ prior to root formation when the tooth was radiographically visible. Anterior and posterior stable structure reference landmarks were marked on pretreatment (T_1_) tracing and transferred to the superimposed post-treatment (T_2_) tracing.

**Figure 2.**
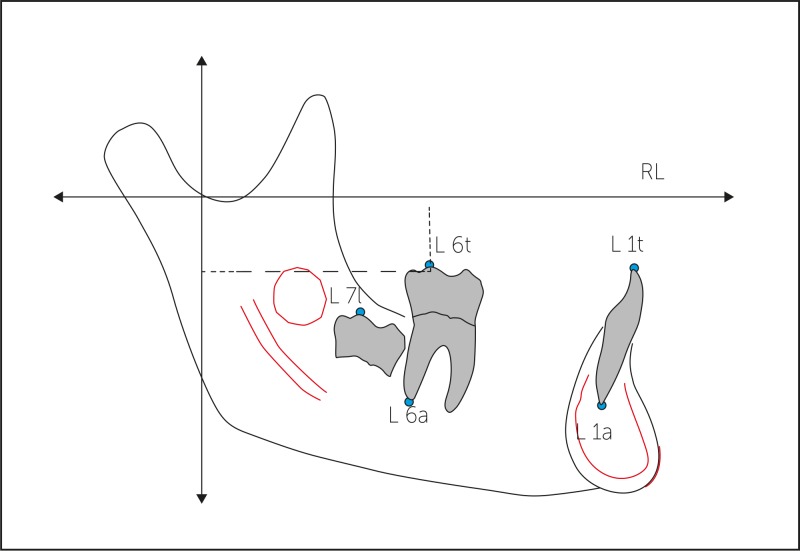
Tracing of mandible showing the five landmarks identified, the stable structures used to superimpose upon (highlighted in red) and the horizontal and vertical reference lines (RL) used for the measurements, with an example of the horizontal and vertical measurements of the molar cusp to the reference lines.

Horizontal and vertical movements of first and second molars, as well as central incisors were assessed in relation to a horizontal reference line (RL) oriented along T_1_ occlusal plane (Fig 2). The anteroposterior changes of first molar crown (L6t, lower molar tip) were measured parallel to RL; the vertical changes were measured perpendicular to RL. Horizontal anterior and vertical superior changes were recorded as positive. All cephalograms were traced and digitized by one investigator using Dentofacial Planner^(c)^ (Dentofacial Software Inc., Toronto, Canada).

Measurements were transferred to SPSS software (version 20.0, SPSS, Chicago, IL, USA) for evaluation. Based on skewness and kurtosis statistics, the variables were judged as normally distributed. Paired t-tests were used to evaluate changes over time in the horizontal and vertical tooth movements due to LB therapy (i.e. differences between pre-treatment and post-treatment). A probability level of 0.05 was used to determine statistical significance.

## RESULTS

**Figure 3.**
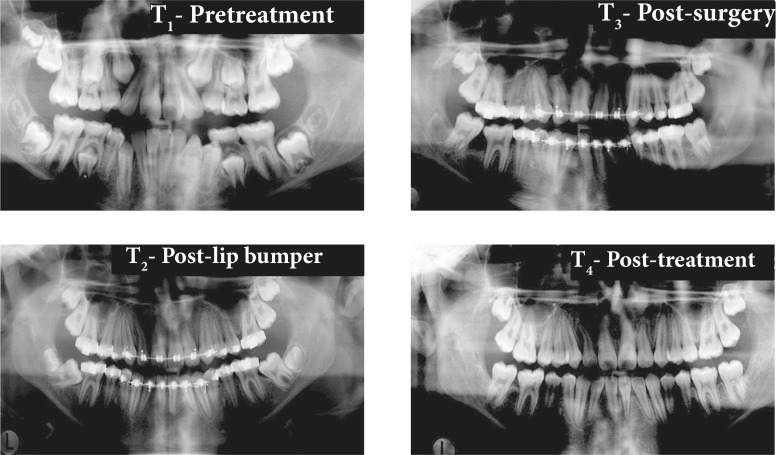
Panoramic radiographs of one of the patients who had second molar impaction. Surgical uncovering treatment of second molar was required.

**Figure 4.**
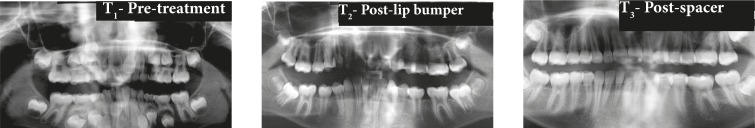
Panoramic radiographs of one of five patients who had second molar impaction. Spacer treatment was performed on this case.

Based on the panoramic radiographs, eight patients (11.9%) had impacted second molars at the end of LB bumper therapy (Table 1). Patients who showed second molars eruption disturbances ranged in age from 8.1 to 12.9 years at the start of LB therapy, and had been treated from 0.8 to 2.9 years. At the end of LB treatment, the roots of second molars were at least 3/4 complete in all but one case. Two patients had fully erupted maxillary molars. Out of eight cases, five (7.5%) showed unilateral second molar impaction while three (4.5%) showed bilateral impaction. Two patients (3%) had bilaterally impacted second molars that required surgical correction (Fig 3). Out of six patients (9%) who had impacted second molars, one was self-corrected and while five were corrected with spacers (Fig 4).

**Table 1.** Description of eight patients who showed second molar impacted at the end of LB therapy.

Id	Age at T_1_ (years)	T_1_-T_2_ (years)	Root length (Second mandibular molar)	Second mandibular molar at T_2_	Impacted	Treatment required	Age at T_2_ >14 years
1	8.1	2.3	3/4	Yes	Left	Spacer	Yes
2	8.4	2.9	7/8	No	Right	Spacer	No
3	8.9	1.9	7/8	No	Left	Self-correction	No
4	9.6	1.0	1/2	No	Bilateral	Surgery	No
5	10.1	4.2	N/A	No	Bilateral	Spacer	No
6	11.4	1.3	7/8	No	Bilateral	Surgery	No
7	11.8	0.8	Full	Yes	Left	Spacer	No
8	12.9	1.4	3/4	No	Right	Spacer	Yes

The mandibular first molar cusp tip and apex migrated forward 1.3 mm and 2.3 mm, respectively, resulting in an apparent tip-back of the first molar (Table 2). Second molar tip showed no statistically significant horizontal movement. Incisor tip moved forward 1.2 mm. Second molars erupted 5.5 mm, while first molar and mandibular incisors approximately erupted 1.5 mm.

**Table 2.** Horizontal and vertical tooth movements due to LB therapy.

	Mean ± SD		Prob.
Horizontal movement			
Mandibular 6 - cusp tip	1.32 ± 1.70		001
Mandibular 6 - apex	2.33 ± 1.73		< 0.001
Mandibular 7 - cusp tip	0.51 ± 2.05		0.232
Mandibular 1 - cusp tip	1.20 ± 1.46		0.001
Mandibular 1 - apex	0.54 ± 1.36		0.061
Vertical movement			
Mandibular 6 - cusp tip	1.68 ± 1.32		< 0.001
Mandibular 6 - apex	1.50 ± 1.44		< 0.001
Mandibular 7 - cusp tip	5.54 ± 2.85		< 0.001
Mandibular 1 - cusp tip	1.45 ± 1.07		< 0.001
Mandibular 1 - apex	1.65 ± 1.22		< 0.001

## DISCUSSION

On average, first molars - especially the root apices - moved mesially during LB treatment. The distal tipping that occurred was due to less forward movement of the cusp than the apex. Also using mandibular superimpositions, previous studies have shown both distal and mesial mandibular first molar movements associated with LB treatment.^6^^,^ 7 ^,^ 8 Nevant et al,^6^ assessing children aged 12.1 years old at the start of treatment, reported distal movement of the crown and mesial movement of the root apex. Based on their cephalometric analysis, Davidovitch et al^8^ reported slight distal molar movement after six months of LB therapy, but movements were not statistically different from control. Werner et al,^7^ who assessed 9.9-year-old patients at the start of treatment, showed that only 12% of LB cases showed distal movement of first mandibular molars (less than 1.5 mm), 58% had no changes and 30% showed mesial movements (maximum of 4 mm) during treatment.

As originally identified by Werner et al,^7^ the difference between horizontal molar movements found in different studies can, at least partially, be related to leeway space. Patients who started treatment in the permanent dentition tend to show distal tipping of first molars, while most of those who started in the late mixed dentition have shown no or little mesial movement of first molars. This indicates that leeway space during the transition from mixed to permanent dentition is not maintained, which helps to explain why the majority of untreated patients showed normal eruption of second molars.

Despite the fact that first molars moved mesially, LB therapy significantly increased the risk of impacting mandibular second molars. Approximately 12% of patients treated with LBs had impacted second molars, which is at least five times greater than what is expected for untreated patients.^10^^-^ 15 Ferro et al^10^ also showed approximately five times the prevalence of second molar impaction among LB treated subjects (7%) when compared to untreated subjects (1.4%). Increased risk of second mandibular molar impaction during LB therapy may be explained in two ways. First, increased distal tipping of first molars has been associated with second molar impaction. The greater the angle between first and second mandibular molars, the greater the risk of second molar impaction.^10^^,^ 12 In addition, individuals undergoing LB treatment usually have anterior mandibular crowding,^6^^,^ 18 ^,^ 19 and a connection between second molar eruption disturbances and crowding have been reported.^12^ Interestingly, the prevalence of impacted second molars and crowding have also increased over time.^12^ Lack of space in the molar region was the primary cause of mesially impacted second molars.^21^

Although LB therapy increases the risk of impaction of second molars, the problem was easily solved. Approximately ⅔ of subjects with second molar impaction were treated with spacers placed between first and second molars, which created space between adjacent teeth and allowed the second molar to erupt into their normal position. Timing of treatment is important. Teeth with eruption disturbances should be treated early (between 11 and 14 years of age), before root formation is complete.^14^^,^ 22 ^,^ 23

There are several options for impacted second molars that cannot be successfully treated with spacers (i.e., tooth extraction, orthodontic uprighting, surgical uprighting, transplantation, surgical-orthodontic approach, and dental implant replacement).^23^^-^ 30 In the present study, surgical exposure of second molar combined with simple orthodontic uprighting mechanics were required for two of the cases. Mesial tipped impacted mandibular second molars show more successful surgical treatment results than vertical or distally tipped molars.^23^ Surgical molar uprighting has proved a predictable and reliable procedure,^31^ and can be performed with or without extraction of the adjacent third molar.^32^ The procedure requires approximately seven months for uprighting and eruption into normal occlusion.^33^ Once more, surgical repositioning is best performed before the roots have completely formed, especially when bodily movement of tooth rather than simple uprighting is required.^34^

While LB will not cause problems in the vast majority of patients, the higher than expected incidence of second mandibular molar impaction makes it necessary to suggest some clinical guidelines. First, lip bumper therapy may not be appropriate for patients with preexisting conditions that increase the risk of impaction. For example, it has been shown that the probability of mandibular second molars impaction is greater among individuals who have: 1) first molars that are closer to the anterior border of the ramus; 2) second molars that are mesially tipped during root formation; 3) shorter mesial than distal second molar root lengths.^12^^,^ 15 Those concerned about the possibility of lip bumper second molar impaction could use a smaller lip bumper (e.g. wire covered with shrink tubing), which does not distally tip the molar back as much as a larger lip bumper with plastic shields.^6^

This observational study has its limitations. Although the literature has shown less than 2.5% of impacted tooth without treatment and our study showed approximately 12%, the lack of a control group could be a potential bias for the results of this study. Randomized control studies are the gold standard, but retrospective observational studies are necessary to start future research. Other potential problem was that RPE treatment could influence buccolingual tipping of posterior lower teeth, and thus affect LB treatment. Therefore, future randomized clinical trials are necessary to substantiate these observational study and others from literature.

## CONCLUSION

1. Lip bumper therapy increases the risk of second molar impaction;
2. First molars tipped back due to lesser amount mesial crown than mesial apex movement;
3. Second molar impaction associated with lip bumper therapy can, in most instances, be easily treated with spacers.
